# The role of musical self-concept and embodiment in stress and flourishing among Chinese university music students

**DOI:** 10.3389/fpsyg.2025.1495005

**Published:** 2025-07-25

**Authors:** Xia Yu, Fumei Xu

**Affiliations:** School of Aviation Service and Music, Nanchang Hangkong University, Nanchang, China

**Keywords:** academic stress, embodiment, musical self-concept, flourishing, Chinese university music students, wellbeing

## Abstract

This study explores the embodied role of musical self-concept in the relationship between academic stress and flourishing among Chinese university music students. Music students face distinctively high levels of academic stress due to intensive practice schedules, performance anxiety, and demanding technical and creative assessments, all of which engage both their physical and psychological selves. Flourishing, defined as the integration of emotional, psychological, and social wellbeing, is essential for positive psychological health. Our sample consisted of 1,767 Chinese university music students. The findings reveal that academic stress has a significant and negative impact on flourishing, consistent with the literature on the harmful effects of stress on well-being. This study further identifies that the embodied nature of musical self-concept, particularly the emotional and communicative dimensions, significantly mediates the relationship between academic stress and flourishing. In contrast, the ability and ambition component did not show a significant mediating effect. The embodied aspects of musical self-concept are evident in the ways students physically engage with their instruments, experience emotions during performance, and integrate their sense of self into their musical practice. These results highlight the importance of cultivating a positive and embodied musical self-concept to counteract the detrimental effects of academic stress and foster flourishing. Interventions aimed at enhancing embodied musical practices and self-awareness may be particularly effective in supporting students’ overall wellbeing. This research contributes to a deeper understanding of the psychological and embodied dimensions that influence the wellbeing of music students, offering practical implications for educational strategies to enhance their flourishing.

## Introduction

Picture a young violinist in a practice room at a Chinese university, her bow poised over the strings. She’s not just preparing for her next recital—she’s navigating a complex world of expectations, dreams, and pressures. This scene, replicated in countless practice rooms across China, captures the essence of our study.

Music students face unique challenges that intertwine the physical, emotional, and cognitive aspects of their lives. Intensive practice schedules demand sustained physical effort, while performance anxiety engages both the body and mind in a deeply embodied response (Wang, 2024). These students are not simply learners—they are performers whose flourishing depends on how their musical self-concept integrates these embodied experiences. Flourishing, in this context, involves not only achieving emotional and psychological balance but also harmonizing the physical demands and rewards of musical practice with their personal and social well-being (Tu, 2022).

The embodied nature of musical self-concept becomes particularly significant in understanding how music students navigate academic stress (Yang and Welch, 2023). Stress is not merely a cognitive burden but an experience that resonates physically, influencing posture, breathing, and overall well-being. Similarly, flourishing is not an abstract concept but a lived, embodied state that reflects students’ holistic engagement with their musical and academic lives (Wang et al., 2023). Recognizing this, we examine how dimensions of musical self-concept—such as communication, ability, ambition, emotion, and spirituality—manifest through students’ embodied practices, shaping their ability to thrive under pressure (Spychiger, 2017).

Our research explores the mechanisms through which musical self-concept mediates the relationship between academic stress and flourishing (Zhang, 2023). By emphasizing embodiment, we aim to explore how physical engagement with music and musicians’ embodied identities impact their psychological well-being and capacity to flourish (Zhang, 2024).

These insights provide a deeper understanding of the interplay between stress, embodiment, and well-being, offering actionable strategies for educators to support students not only in their technical growth but also in their holistic development (Yunchao and Kamarudin, 2024).

By situating embodiment at the center of this inquiry, we hope to advance music education practices that acknowledge the physical, emotional, and psychological integration essential to a positive musical self-concept. These findings promise to enhance student support services and inform institutional policies, helping music students not just survive but thrive in the demanding world of Chinese higher education.

## Literature review and hypotheses development

### Conceptual framework and key definitions

In this study, we focus on three core psychological constructs—academic stress, flourishing, and embodiment—as well as the multidimensional nature of musical self-concept. We provide operational definitions below, along with references to seminal literature that informs our theoretical framing.

Academic stress is defined, following Lazarus and Folkman’s (1984) transactional theory of stress, as a psychological state that arises when individuals perceive that environmental demands exceed their available coping resources. In the context of university students, this often manifests as tension, anxiety, and fear of failure related to academic tasks, expectations, and evaluations (LeBlanc et al., 1997). For music students, these stressors are compounded by the demands of performance, competitive environments, and prolonged practice routines.

Flourishing, as conceptualized by Diener et al. (2010) and elaborated in Seligman’s (2011) PERMA model, refers to a state of optimal psychological functioning characterized by positive emotion, engagement, relationships, meaning, and accomplishment. Within music education, flourishing also encompasses creative expression, emotional resilience, and the alignment of personal goals with artistic identity (Ascenso et al., 2018).

Embodiment is understood here through the lens of Johnson (Johnson, 2007), Leman (2007), and (Zbikowski, 2017), who view cognition, identity, and meaning as grounded in bodily experience. In music, embodiment is reflected in sensorimotor engagement with instruments, the physical-emotional resonance of sound, and the integration of musical gestures into the formation of identity. Music students’ embodied experiences shape how they interpret stress and build pathways to well-being.

Musical self-concept is defined as an individual’s self-perception about their musical abilities, experiences, and identity (Petersen and Camp, 2016; Schmidt et al., 2006). We adopt a multidimensional perspective, distinguishing between:

- Instrumental self-concept: the technical, skill-based, and achievement-oriented aspects of one’s musical identity (e.g., ability, ambition, discipline). In this study, instrumental self-concept refers to students’ self-perceived technical competence, achievement orientation, and musical ambition. It reflects how confident they feel in their ability to meet demanding performance standards, pursue musical excellence, and set high goals in their instrumental or vocal development. It does not refer to communication through music—that is captured separately by the communication self-concept dimension.
- Emotional-spiritual self-concept: the expressive, affective, and transcendent engagement with music, encompassing emotional resonance, meaning-making, and self-integration through musical practice.

These dimensions are grounded in research using the Musical Self-Concept Inquiry (MUSCI; Spychiger, 2017) and form the theoretical basis for our mediation model, which links stress to flourishing.

### Academic stress among Chinese music students and its direct relationship with students’ flourishing

University music students often experience academic stress through a unique combination of artistic, technical, and educational pressures. Unlike students in more conventional academic fields, their curriculum typically includes performance-based evaluations (e.g., juries, solo recitals, ensemble participation), extensive hours of solitary practice, and high expectations from teachers and peers. Perfectionist standards, competitive admission environments, and a strong identification with musical success often exacerbate these issues. Furthermore, many music students must meet the demands of general academic coursework while simultaneously preparing for demanding artistic outputs. As a result, the “academic” stress experienced by music students reflects both the conventional pressures of university life and the emotional, physical, and identity-based challenges intrinsic to music performance and training.

Academic stress is a multifaceted phenomenon experienced by students, characterized by tension, anxiety, and pressure related to academic tasks and responsibilities. This stress can stem from high expectations, workload, and the fear of failure. In the context of this study, our focus is on individuals participating in formal university-level training that integrates academic requirements and musical mastery. Therefore, academic stress refers not only to traditional scholarly demands—such as coursework, assessments, and institutional expectations—but also to discipline-specific pressures inherent to music education, including performance anxiety, juried evaluations, and intensive technical preparation. This integrative academic environment creates a distinct and multifaceted form of stress for music students. Music students experience particularly high levels of academic stress due to the unique demands of their curriculum, which includes intensive practice schedules, performance anxiety, and rigorous technical and creative assessments (Baiyan et al., 2024). In this context, academic skills encompass both general and discipline-specific competencies. General academic skills include time management, critical reading, and academic writing. More importantly for music students, discipline-specific skills include the application of music theory, score analysis, structured practice planning, and reflective performance evaluation. These skills require high levels of self-regulation and cognitive integration, which are essential for the development of a musical self-concept and academic success.

Factors influencing academic stress in music students include performance anxiety, the pressure to achieve high standards, and intense competition. Research indicates that music students often experience higher stress levels compared to their peers in other disciplines due to these unique challenges (LeBlanc et al., 1997). In China, academic stress is heightened by cultural and educational factors that place a premium on academic achievement and success, often conducting to severe health problems as well as suicidal ideation among younger (Wang et al., 2023). Chinese students often face intense pressure from family expectations and societal norms. The Confucian heritage culture, which emphasizes hard work, perseverance, and respect for authority, significantly shapes the educational experiences of Chinese students, leading to heightened stress levels as they strive to meet high expectations (Li et al., 2023; Sun et al., 2013). The combination of these cultural expectations and the intrinsic demands of music education creates a uniquely challenging environment.

### Flourishing

Flourishing, in this context, involves not only achieving emotional and psychological balance but also harmonizing the physical demands and rewards of musical practice with their personal and social well-being (Tu, 2022). Similar insights are echoed in the work of Ascenso et al. (2018), who explored flourishing in professional classical musicians and emphasized the multidimensional nature of well-being in music careers, including emotional resilience, autonomy, and a sense of purpose derived from musical engagement. This aligns with Seligman’s (2011) PERMA model, which defines flourishing through five core elements: Positive Emotion, Engagement, Relationships, Meaning, and Accomplishment (Seligman, 2011).

In university students, flourishing involves thriving academically and personally, demonstrating resilience, and engaging in meaningful activities (Li Y. et al., 2020). For music students, flourishing can manifest in creative achievements, personal growth, and fulfilling social connections within their academic environment (Volpe, 2021). For university-level music students in China, flourishing entails navigating the dual pressures of academic and musical excellence while maintaining overall well-being (Howell and Buro, 2015). This includes achieving high standards, personal fulfillment, resilience, and a sense of community within their academic setting. Cultural values that prioritize collective well-being, harmony, and academic success influence Chinese students’ concept of flourishing (Li B. et al., 2020). Meeting familial and societal expectations can either contribute to or detract from their wellbeing, depending on individual circumstances and coping mechanisms.

The relationship between academic stress and flourishing is complex. Academic stress, while sometimes serving as a motivator, is generally detrimental when chronic or excessive, impeding students’ ability to flourish (Mawang et al., 2019). For students enrolled in Chinese music degree programs, high-stress levels can undermine psychological well-being, leading to burnout, anxiety, and depression, which hinder flourishing (Hruska, 2019). In the Chinese cultural context, where familial and societal expectations often intensify academic pressures, understanding how students manage stress becomes especially relevant (Bailey, 2023). Effective stress management has the potential to foster resilience and self-efficacy—key factors that support flourishing. Investigating this relationship may inform strategies to cultivate academic and emotional well-being in higher music education (Jääskeläinen, 2023).

### The mediating role of musical self-concept in two ways: instrumental and emotional

Musical self-concept is a vital construct in understanding the relationship between academic stress and flourishing among music students. It encapsulates a person’s answers to the questions “who I am” and “what I can do” about music (Spychiger, 2017). According to Bernecker et al. (2006), musical self-concept encompasses various facets of ideas, perceptions, and assessments—a collection of cognitions regarding one’s musical activities. In conclusion, musical self-concept is considered a domain of an individual’s self-concept and identity, particularly significant for musicians. Despite the construct’s widely accepted definition (Ruismäki and Tereska, 2006), the factor structure of the initial measuring tool (Fiedler and Spychiger, 2017), originally developed in German and translated into English, showed avenues for improvement. Further research attempted to adapt the measurement to Chinese students (Petersen and Camp, 2016), providing a new factor structure but based only on a few dance students. Given the relevance of musical self-concept to music development and performance, the present study aims to provide empirical evidence specifically focused on Chinese university music students. To enhance cultural and linguistic alignment, we used a shortened form of the MUSCI, selecting one item with the highest factor loading per subdimension based on prior adaptation studies.

Previous studies have shown that, on the one hand, musical education exerts an effect on self-concept (Dege et al., 2014), practice of a musical instrument promotes self-concept among adolescents and further performance achievement (Guhn et al., 2020), motivation (Qarri, 2023), and global musical experience (Schmidt et al., 2006). On the other hand, empirical research also supported the relationships between musical self-concept and optimism (Moore et al., 2003), subjective well-being, and participation in musical academic activities (Austin, 1988), suggesting a hinge role of musical self-concept between antecedents and consequences (Janurik et al., 2023). Examples of musical academic activities include music theory examinations, ensemble rehearsals, individual performance assessments (e.g., juries or recitals), sight-reading tests, coursework in music history and analysis, and the preparation of written assignments or research projects related to musical practice. Hence, the musical self-concept could serve as a mediator in the direct relationships between academic stress and flourishing through two primary pathways: Instrumental Self-Concept and Emotional-Spiritual Self-Concept.

Instrumental Self-Concept refers to a musician’s perception of their technical abilities and competencies with their instrument or voice. This facet includes communication and the ability and ambition to express ideas and emotions through music and to achieve musical excellence. Communication involves the capacity to convey emotions, thoughts, and stories through musical performance. Ability and ambition refer to the perceived skill level and the drive to achieve musical excellence. Research suggests that a strong instrumental self-concept can buffer the negative effects of academic stress by fostering a sense of competence and control. Music students who view themselves as capable and ambitious are more likely to thrive despite the pressures they face (Zarza-Alzugaray et al., 2024).

Emotional-Spiritual Self-Concept encompasses a musician’s emotional connection to music and its role in their spiritual life (Petrides et al., 2006). This dimension includes the emotional satisfaction and catharsis derived from musical activities and the sense of transcendence and deeper meaning associated with musical engagement (Fiedler and Spychiger, 2017; Petersen and Camp, 2016). A growing body of research suggests that emotional and spiritual engagement in musical activities may contribute positively to students’ well-being by fostering emotional regulation, meaning-making, and resilience (Asmus and Harrison, 1990). In this sense, a strong emotional-spiritual self-concept may serve as a psychological resource that helps music students navigate stress and sustain flourishing in demanding academic contexts (Chen, 2024).

In this study, we adopt a mediation framework to examine how academic stress influences flourishing through the construct of musical self-concept. Mediation is conceptualized as a psychological mechanism through which external stressors impact well-being indirectly, by altering internal beliefs, attitudes, or self-perceptions (Baron and Kenny, 1986; Hayes, 2013). Specifically, we propose that two dimensions of musical self-concept—instrumental and emotional-spiritual—act as mediators that shape how students interpret and respond to academic stress. These self-representations are not static traits but dynamic cognitive-affective systems influenced by performance experience, identity development, and emotional regulation. Thus, musical self-concept serves as the internal lens through which stress is filtered and either amplified or neutralized in its impact on flourishing. Understanding these facets of musical self-concept is crucial for comprehending how music students navigate the dual pressures of academic and musical excellence while maintaining their overall well-being. By focusing on these mediating factors, educators and policymakers can better support music students in achieving both academic success and personal flourishing.

### Hypotheses statement

#### Direct effects hypotheses

*H1*: There is a significant direct effect of Music Students’ Academic Stress (X) on Music Students’ Flourishing (Y).

Based on the above-revised conceptualization, distinguishing between a more instrumental side of the musical self-concept and a more emotional-spiritual self-concept, the following differentiated pathways of influences are hypothesized:

#### Indirect effects hypotheses through instrumental self-concept

*H2*: Music Students’ Academic Stress (X) has a significant indirect effect on Music Students’ Flourishing (Y) through Musical Self-concept Communication (M1).

*H3*: Music Students’ Academic Stress (X) has a significant indirect effect on Music Students’ Flourishing (Y) through Musical Self-concept Ability and Ambition (M2).

#### Indirect effects hypotheses through emotional-spiritual self-concept

*H4*: Music Students’ Academic Stress (X) has a significant indirect effect on Music Students’ Flourishing (Y) through Musical Self-concept Emotion (M3).

*H5*: Music Students’ Academic Stress (X) has a significant indirect effect on Music Students’ Flourishing (Y) through Musical Self-concept Spirituality (M4).

#### Combined mediator effects hypotheses

*H6*: Music Students’ Academic Stress (X) has a significant indirect effect on Music Students’ Flourishing (Y) through the combined mediating effects of Musical Self-concept Communication (M1) and Musical Self-concept Ability and Ambition (M2).

*H7*: Music Students’ Academic Stress (X) has a significant indirect effect on Music Students’ Flourishing (Y) through the combined mediating effects of Musical Self-concept Emotion (M3) and Musical Self-concept Spirituality (M4).

**Figure 1:**
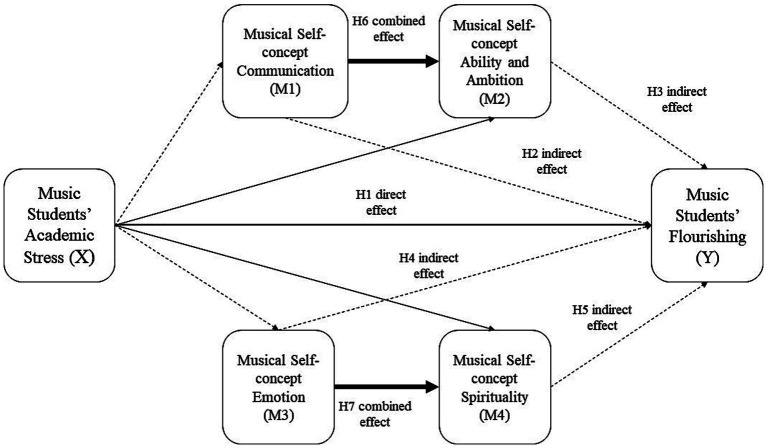
Full set of hypotheses.

Figure 1 depicts the full set of Hypotheses.

## Methods

### Participants

The sample of Chinese university music students consists of 1,767 individuals. Within this group, females represent 57.3% (1,013 students), while males account for 42.7% (754 students), reflecting a slightly higher proportion of female students in this field. Among them, 43.8% are pursuing or have completed college-level studies, 41.6% are engaged in master’s programs, and 14.6% are involved in doctoral studies. This distribution shows a significant proportion of students advancing beyond undergraduate education, indicative of a commitment to higher-level academic and professional training in music. The age profile of the sample has an average of 25.33 years, with a standard deviation of 5.214, suggesting a range of ages typical of both graduate and undergraduate populations. The ages vary from 18, likely representing younger undergraduates, to 37, which may include more seasoned doctoral candidates or those who have taken non-traditional paths through their education. This data reflects a broad engagement in music studies across various stages of higher education, demonstrating the students’ long-term dedication to their field. The representation across different educational levels highlights the depth and diversity of musical education and training among the sample. In this study, the term *university music students* refers to individuals enrolled in formal higher education programs in music at various types of Chinese institutions, including conservatories, normal universities, and comprehensive universities. These programs span multiple subfields such as instrumental and vocal performance, music education, composition, conducting, and musicology. This diversity reflects the broad academic and artistic training paths represented in the sample.

### Procedure

**Study Design:** This cross-sectional study was designed to investigate the mediating role of musical self-concept in the relationship between academic stress and flourishing among music students in Chinese higher education. In addition, it aimed to capture a snapshot of the students’ demographic and academic characteristics, including age and educational level, to provide context for interpreting the psychological constructs under study and to ensure the sample’s relevance to the broader population of music students in higher education.

**Ethical Considerations:** The study was conducted in accordance with the ethical guidelines stipulated in the Declaration of Helsinki and its later amendments, emphasizing ethical principles for research involving human subjects. The Ethical Committee of the Nanchang Hangkong University approved the project previously. All participants were provided with an informed consent form before participating in the survey, which detailed the purpose of the research, the voluntary nature of their participation, and the confidential handling of their data. This form needed to be completed and submitted electronically before they could proceed with the survey by clicking the positive answer to the first three questions of the questionnaire. To ensure data protection and privacy, all personal information that could identify the participants was omitted from the dataset. The collected data were encrypted and securely stored on Nanchang Hangkong University’s servers, accessible only to the research team for analysis purposes. They will be retained for a period as specified by university guidelines before secure disposal.

**Data Collection:** The data were collected using a structured online survey disseminated through popular social networking platforms in China, such as WeChat and Weibo, to reach a broad audience of university students currently enrolled in music programs. The survey was hosted on SurveyMonkey, a commercial survey tool known for its robust data collection and analysis capabilities.

**Survey Conduction:** The survey was conducted over 3 smonths during the first semester of 2024, allowing sufficient time for participants to respond. The questions focused on gathering specific data about the student’s educational level and age, ensuring concise and relevant information was obtained. All instruments, including demographic questions, ESSA, MUSCI, and the Flourishing Scale (FS), were embedded in a single online questionnaire administered via SurveyMonkey in one data collection phase.

### Instruments

To better reflect the embodied aspects of musical self-concept, we interpret the MUSCI dimensions—particularly Communication, Emotion, and Spirituality—as operationalizing students’ physical and emotional engagement with music. For instance, the Communication dimension captures the embodied experience of interacting with others through performance, while Emotion and Spirituality reflect internalized physical-emotional states activated during musical engagement.

*Music Students’ Academic Stress: The Music Students’* Academic Stress was measured using a reduced version of the Educational Stress Scale for Adolescents (ESSA). The ESSA is a 16-item self-report measure that evaluates students’ perceived educational stress within the school environment. For this study, a condensed version was employed, which includes one item per dimension, selected based on the highest factor loading in each domain. This approach ensures that the most representative and impactful items are utilized for a focused assessment.

Participants responded to these items on a 5-point Likert scale, ranging from 1 (Strongly disagree) to 5 (Strongly agree). This scale captures the intensity of stress across five key domains: pressure from study, worry about grades, self-expectation, workload, and despondency. The following items were used in the reduced ESSA for this study: (Pressure from Study) “I feel a lot of pressure in my daily studying.”; (Worry about Grades) “I feel that I have disappointed my parents when my test/exam results are poor.”; (Self-Expectation), “I feel stressed when I do not live up to my standards; (Workload), “I feel there is too much homework” and (Despondency), “Future education and employment bring me a lot of academic pressure.”

*Musical Self-concept* dimensions were measured using the Musical Self-Concept Inquiry (MUSCI), a tool specifically designed for musicians, including those currently playing an instrument or using their voice. The MUSCI was originally developed for adult populations and has been validated in samples of younger children and secondary education students (Spychiger, 2017). The original MUSCI was developed by Spychiger (2017) as a multidimensional scale composed of seven first-order factors—such as Ability and Ambition, Emotion, Communication, Spirituality, Musical Identity, Social Relations, and Self-Regulation—which capture various aspects of musical self-concept. The scale was validated using exploratory and confirmatory factor analysis on samples ranging from secondary students to adult musicians. For the present study, we focused on four core dimensions—Communication, Ability and Ambition, Emotion, and Spirituality—based on their theoretical alignment and empirical strength. From each subscale, three items with the highest factor loadings in prior studies were selected to create a shortened, psychometrically robust version of the scale. Participants responded to each item on a five-point Likert scale ranging from 1 (fully disagree) to 5 (fully agree).

*Musical Self-concept in Communication (M1)* evaluates the extent to which individuals use music as a medium for communication. This dimension included three items that capture various aspects of musical communication, such as the ease of performing on stage and the ability to connect with the audience. For example, one of the items in this dimension is “I play music in order to communicate with other people.” Other items include statements like “I easily become part of a musical ensemble” and “I sense that the music I perform connects people,” highlighting different facets of musical communication.

*Ability and Ambition (M2)* focuses on the participants’ perceptions of their musical capabilities and aspirations. This dimension consists of three items that assess self-perceived musical proficiency and the drive to achieve high musical standards. An example item from this dimension is “I am capable of achieving my musical goals.” Additional items include “I am musically ambitious” and “I strive toward high musical achievement,” reflecting the students’ ambitions and self-assessed abilities in their musical endeavors.

*Emotion (M3)* measures the emotional aspects of musical involvement. This dimension includes four items that explore how musical activities impact the students’ emotions and how they use music to express their feelings. Music can affect students’ emotional states in multiple ways: through the modulation of mood, the channeling of emotional expression, and the generation of affective resonance during performance or listening. Engaging in musical activities allows students to process complex feelings, experience catharsis, and cultivate emotional awareness—functions that are particularly relevant in emotionally demanding academic environments. This emotional engagement, in turn, is a key component of their self-concept as musicians. For instance, the item “Musical activity can alter my mood” exemplifies this dimension. Other items, such as “When making music, I have to be able to forget time and place” and “The music in which I am involved impacts my emotions,” further illustrate how music affects the emotional states of the students.

*Spirituality (M4)* assesses the spiritual experiences and purposes associated with musical activities. This dimension comprises three items that examine the role of music in eliciting spiritual experiences and fostering a sense of the divine. One of the items in this dimension is, “For me, music-making is a special kind of prayer.” Additional items include “I make music to feel the divine” and “With my music, I can elicit change in people,” highlighting the spiritual and transformative aspects of musical engagement.

The adapted MUSCI used in this study consisted of three representative items per subdimension, selected for their high factor loading from prior validation research. Despite this shortened format, the scales’ reliability ranged from Cronbach’s Alphas of 0.89 in the Communication dimension to 0.69 in the Spirituality dimension, 0.69 in the Ability and Ambition dimension, and 83 in the Emotion dimension.

*Music Students Flourishing*: The flourishing of music students was assessed using the Simplified Chinese Version of the Flourishing Scale (FS), an 8-item instrument designed to evaluate various aspects of human functioning. The scale is based on the previous version (Diener et al., 2010) and adapted to the Chinese population (Tang et al., 2016). Participants rated each item on a 7-point Likert scale, ranging from 1 (strongly disagree) to 7 (strongly agree). A higher mean score on this scale indicates a greater degree of well-being and flourishing across key areas of functioning. The FS has demonstrated robust psychometric properties across diverse cultural contexts, with validation studies confirming its reliability and validity in various countries (Diener et al., 2010; Hone et al., 2014; Silva and Caetano, 2013), while the adapted version also provides a comprehensive measure of respondents’ overall wellbeing, capturing essential elements of flourishing and high functioning in life. Sample items from the scale include: “I lead a purposeful and meaningful life,” “My social relationships are supportive and rewarding,” and “I actively contribute to the happiness and well-being of others.” These items reflect the multidimensional nature of flourishing, covering aspects such as purpose, engagement, social connection, and self-perceived growth.

### Data analyses

The analysis utilized IBM SPSS Statistics software (version 29.0) to conduct both descriptive and inferential statistical analyses. Pearson’s correlation coefficients were calculated to examine the bivariate relationships among academic stress, musical self-concept dimensions, and flourishing prior to conducting the mediation analysis. Descriptive statistics calculated included means and standard deviations for the variables “Music Students Academic Stress” (X), “Music Students Flourishing” (Y), and the mediators: Musical Self-concept in Communication (M1), Ability and Ambition (M2), Emotion (M3), and Spirituality (M4). These metrics provided an initial understanding of the data characteristics. The mediation analysis was conducted using Process Model 82, facilitated by the PROCESS macro in SPSS (Hayes, 2022).

We conducted mediation analysis using regression-based procedures to estimate both direct and indirect effects of academic stress on flourishing, with musical self-concept dimensions serving as mediators. Flourishing, academic stress, and the four self-concept dimensions (Communication, Emotion, Ambition, Spirituality) were treated as observed variables, based on composite scores from their respective scales. Mediation analysis was selected because it allows us to test the theoretical assumption that musical self-concept functions as a psychological mechanism that transmits the effect of academic stress onto flourishing. This approach goes beyond simple correlation by clarifying potential indirect pathways, thereby offering a more precise interpretation of how specific self-concept dimensions contribute to students’ well-being.

This model is adept at assessing both direct and indirect effects of an independent variable (X) on a dependent variable (Y) through multiple mediators (M1, M2, M3, M4) configured in a serial sequence. The utilization of this model allowed for an intricate examination of how Academic Stress influences Flourishing via various dimensions of Musical Self-concept. The significance of indirect effects was tested using a bootstrapping procedure with 5,000 resamples. This approach is implemented through the PROCESS macro and does not depend on normality assumptions, making it ideal for our mediation analysis. Confidence intervals (CI) were calculated at the 95% level, with statistical significance indicated by intervals that do not contain zero. The 95% confidence intervals obtained from the bootstrapping are crucial for determining the statistical significance of the model’s mediation effects. This method effectively reduces the risk of Type I errors, providing a more reliable indication of significant mediation pathways.

## Results

### Descriptive statistics and Pearson’s correlations

The means and standard deviations, presented in Table 1, indicate the central tendency and variability of these constructs among the sample of music students.

**Table 1:** Descriptive statistics and Pearson’s correlations.

Variable	*M*	SD	1	2	3	4	5	6
1. Music students’ academic stress (X)	2.44	1.11	--					
2. Musical self-concept communication (M1)	3.60	0.97	−0.064**	--				
3. Musical self-concept ability and ambition (M2)	3.86	0.71	−0.019	0.428**	--			
4. Musical self-concept emotion (M3)	3.53	0.79	−0.116**	0.600**	0.491**	--		
5. Musical self-concept spirituality (M4)	3.60	0.89	−0.161**	0.404**	0.436**	0.620**	--	
6. Music students’ flourishing (Y)	3.44	0.84	−0.258**	0.508**	0.334**	0.653**	0.602**	--

**p <0.01.

Pearson’s correlation coefficients were calculated to examine the relationships between these variables. The results indicate statistically significant but weak negative correlations (r < 0.3) between Music Students’ Academic Stress and all other variables, except for Musical Self-Concept Ability and Ambition. This suggests that while academic stress is meaningfully associated with lower levels of flourishing and most dimensions of musical self-concept, the strength of these relationships is modest. Musical Self-concept Communication (M1) showed significant positive correlations with Musical Self-concept Ability and Ambition (M2), Musical Self-concept Emotion (M3), Musical Self-concept Spirituality (M4), and Music Students’ Flourishing (Y). These correlations suggest that students who perceive themselves as better communicators in music also tend to have higher abilities and ambitions, stronger emotional connections, a deeper spirituality in music, and overall greater flourishing. Musical Self-concept Ability and Ambition (M2) also showed significant positive correlations with Musical Self-concept Emotion (M3), Musical Self-concept Spirituality (M4), and Music Students’ Flourishing (Y). This suggests that students who feel more capable and ambitious in music also experience higher emotional and spiritual connections to music and greater overall flourishing. Musical Self-concept Emotion (M3) and Musical Self-concept Spirituality (M4) were strongly positively correlated with each other and with Music Students’ Flourishing (Y). These findings highlight the intertwined nature of emotional and spiritual self-concepts in music and their collective contribution to students’ overall flourishing.

Overall, the correlation analysis reveals a complex interplay between academic stress, various aspects of musical self-concept, and students’ flourishing.

### Hypotheses testing

#### Direct effects

*H1*: The results confirm that Music Students’ Academic Stress has a significant negative direct effect on Music Students’ Flourishing (𝛽= − 0.157, 𝑝<0.001, 95% CI [−0.143, −0.094]). The model predicting Music Students’ Flourishing—based on academic stress and all four musical self-concept dimensions—explains 53.4% of the variance (𝑅^2^ = 0.534), as calculated from the SPSS output. This overall *R*^2^ value pertains to the final regression equation including all mediators and is not separately listed in Table 2.

**Table 2:** Direct effects of predictors on outcome variables.

Predictor	*B*	SE	*t*	*p*	LLCI	ULCI	*β*
Outcome variable: musical self-concept communication							
Music students’ academic stress	−0.056	0.021	−2.684	0.007	−0.096	−0.015	−0.064
Outcome variable: musical self-concept ability and ambition							
Music students’ academic stress	0.005	0.014	0.372	0.710	−0.022	0.032	0.008
Outcome variable: musical self-concept emotion							
Music students’ academic stress	−0.083	0.017	−4.924	0.000	−0.116	−0.050	−0.116
Outcome variable: musical self-concept spirituality							
Music students’ academic stress	−0.072	0.015	−4.806	0.000	−0.101	−0.043	−0.090
Outcome variable: music students’ flourishing							
Music students’ academic stress	−0.119	0.013	−9.480	0.000	−0.143	−0.094	−0.157
Musical self-concept Communication	0.156	0.018	8.706	0.000	0.121	0.192	0.180
Musical self-concept ability and ambition	−0.069	0.023	−2.990	0.003	−0.114	−0.024	−0.058
Musical self-concept emotion	0.390	0.026	15.171	0.000	0.339	0.440	0.368
Musical self-concept spirituality	0.286	0.020	14.156	0.000	0.246	0.325	0.302

B = Unstandardized coefficients; *β* = standardized coefficients; LLCI = 95% Lower Level Confidence Interval; ULCI = 95% Upper-Level Confidence Interval.

#### Indirect effects through instrumental self-concept

*H2*: Music Students’ Academic Stress (X) has a significant indirect effect on Music Students’ Flourishing (Y) through Musical Self-concept Communication (M1). The results indicate a significant indirect effect through Musical Self-concept Communication (𝛽= − 0.009, 95% CI [−0.017, −0.002]), as Table 3 shows.

**Table 3:** Indirect effects of music students’ academic stress on music students’ flourishing.

Path	Effect	BootSE	Boot LLCI	Boot ULCI
Total indirect effect	−0.077	0.014	−0.105	−0.051
Ind1: Music students’ academic stress - > M1 - > Y	−0.009	0.004	−0.017	−0.002
Ind2: Music students’ academic stress - > M2 - > Y	0.000	0.001	−0.003	0.002
Ind3: Music students’ academic stress - > M3 - > Y	−0.032	0.008	−0.048	−0.018
Ind4: Music students’ academic stress - > M4 - > Y	−0.021	0.005	−0.031	−0.011
Ind5: Music students’ academic stress - > M1 - > M2 - > Y	0.001	0.001	0.000	0.003
Ind6: Music students’ academic stress - > M3 - > M4 - > Y	−0.016	0.004	−0.024	−0.009

Ind1: Music Students Academic Stress- > Musical Self-concept Communication- > Music Students Flourishing; Ind2: Music Students Academic Stress- > Musical Self-concept ability and ambition- > Music Students Flourishing; Ind3: Music Students Academic Stress- > Musical Self-concept emotion- > Music Students Flourishing; Ind4: Music Students Academic Stress - > Musical Self-concept spirituality- > Music Students Flourishing; Ind5: Music Students Academic Stress - > Musical Self-concept Communication- > Musical Self-concept ability and ambition- > Music Students Flourishing; Ind6: Music Students Academic Stress - > Musical Self-concept emotion- > Musical Self-concept spirituality- > Music Students Flourishing.

*H3*: Music Students’ Academic Stress (X) has a significant indirect effect on Music Students’ Flourishing (Y) through Musical Self-concept Ability and Ambition (M2). The results do not indicate a significant indirect effect through Musical Self-concept Ability and Ambition (*β* = 0.000, 95% CI [−0.003, 0.002]).

Table 3 presents the indirect effects of academic stress on flourishing through various musical self-concept dimensions. The total indirect effect is negative and statistically significant (−0.077), suggesting that academic stress reduces flourishing, primarily through its impact on self-concept. Among the individual paths, the strongest mediators are the emotional (Ind3: −0.032) and spiritual (Ind4: −0.021) self-concept dimensions, indicating that when stress diminishes, students’ emotional or spiritual musical engagement, their well-being suffers more noticeably. Communication (Ind1: −0.009) also plays a mediating role, albeit more modestly. In contrast, the ambition/ability dimension (Ind2) does not show a significant mediating effect. Two sequential mediation paths (Ind5 and Ind6) were also tested, with Ind6 (emotion → spirituality) showing a small but significant effect (−0.016), reinforcing the interconnected role of emotional and spiritual engagement in shaping flourishing outcomes.

#### Indirect effects through emotional-spiritual self-concept

*H4*: Music Students’ Academic Stress (X) has a significant indirect effect on Music Students’ Flourishing (Y) through Musical Self-concept Emotion (M3). The results indicate a significant indirect effect through Musical Self-concept Emotion (𝛽= − 0.032, 95% CI [−0.048, −0.018]).

*H5*: Music Students’ Academic Stress (X) has a significant indirect effect on Music Students’ Flourishing (Y) through Musical Self-concept Spirituality (M4). The results indicate a significant indirect effect through Musical Self-concept Spirituality (𝛽= − 0.021, 95% CI [−0.031, −0.011]).

#### Combined mediator effects

*H6*: Music Students’ Academic Stress (X) has a significant indirect effect on Music Students’ Flourishing (Y) through the combined mediating effects of Musical Self-concept Communication (M1) and Musical Self-concept Ability and Ambition (M2). The results indicate that the combined indirect effect through Musical Self-concept Communication and Musical Self-concept Ability and Ambition is not significant (𝛽 = 0.001, 95% CI [0.000, 0.003]).

*H7*: Music Students’ Academic Stress (X) has a significant indirect effect on Music Students’ Flourishing (Y) through the combined mediating effects of Musical Self-concept Emotion (M3) and Musical Self-concept Spirituality (M4). The results confirm a significant indirect effect through the combined mediators of Musical Self-concept Emotion and Musical Self-concept Spirituality (*β* = −0.016, 95% CI [−0.024, −0.009]). In addition to the specified mediating pathways, the total indirect effect of musical self-concept as a composite construct was also found to be significant (*β* = −0.077, 95% CI [−0.105, −0.051]; see Table 3). While not hypothesized explicitly, this result suggests that the collective influence of musical self-concept’s dimensions meaningfully attenuates the impact of academic stress on flourishing. We interpret this as further support for the multidimensional and embodied nature of musical self-concept in supporting students’ well-being.

## Discussion

The present research aimed to examine how musical self-concept—understood as an embodied psychological construct—mediates the relationship between academic stress and flourishing among Chinese university music students. The findings confirmed that specific dimensions of musical self-concept, especially those related to emotional and communicative experiences, significantly buffer the effects of stress. These results support the view that musical flourishing is not solely a cognitive process but one grounded in embodied musical practices.

### Direct effects of music students’ stress on personal wellbeing

Our findings reveal that academic stress has a significantly negative impact on the flourishing of music students, aligning with existing literature that highlights the detrimental effects of stress on students’ well-being. A comparison with previous research helps us underscore the broader implications and suggest potential interventions to mitigate the adverse effects of academic stress on music students. Several studies support the conclusion that academic stress negatively impacts students’ flourishing and overall well-being. For instance, Üstün (2016) found that high levels of academic stress among high school students were associated with lower test performance and increased anxiety, reflecting the broader impact of stress on academic and personal outcomes. These findings open the door to applied implications. If specific aspects of musical self-concept can buffer academic stress, then targeted interventions based on musical engagement may help regulate physiological and psychological responses. Similarly, Laohawattanakun et al. (2011) demonstrated that music training could reduce cortisol levels in students experiencing examination-induced stress, suggesting that targeted interventions might alleviate stress and promote well-being. Further studies observed that listening to relaxing music while studying improved students’ mood and academic performance, suggesting its therapeutic potential in educational settings. However, these effects have been observed primarily among non-music student populations. For music students, whose academic demands are deeply intertwined with music performance and evaluation, music may not always function as a stress-relieving medium and may, in some cases, reinforce academic pressure (Bellier et al., 2020; Gallego-Gómez et al., 2020; Hu et al., 2021). Our findings suggest that musical self-concept—particularly its emotional and spiritual components—may serve as potential targets for future interventions aimed at reducing academic stress and enhancing flourishing among music students. Recent studies emphasized the complex relationship between occupational stress, passion, and well-being among musicians, indicating that stress management strategies tailored to the unique challenges faced by music students are crucial (Portía et al., 2021). Similarly, Deng and Ko (2023) highlighted that reducing academic stress could enhance individual thinking ability among university music students, further supporting the need for effective stress management practices.

### Indirect effects through instrumental self-concept

The study reveals significant indirect effects for the Emotional, Spiritual, and Communicative dimensions of musical self-concept. In contrast, no significant mediation was observed for the Ability and Ambition dimension or the combined instrumental pathway, suggesting that technical confidence alone may not buffer academic stress in the same way as more affective or expressive dimensions. Previous studies support the notion that self-concept plays a crucial role in mediating the relationship between academic stress and flourishing among music students. For instance, Moore and Wilhelm (2019) highlighted that music therapy students’ engagement in self-care practices, including self-awareness, has a significant impact on their perceived stress levels. Higher engagement in self-awareness practices was associated with lower perceived stress, underscoring the importance of self-concept in stress management. Similarly, other studies have found that music education has a positive impact on self-efficacy and stress management skills among undergraduate students, indicating that enhancing self-concept can mitigate the negative effects of academic stress and promote flourishing (Üstün, 2016).

Contrary to the positive impacts of self-concept on managing academic stress, some studies suggest that the relationship may not be straightforward. For example, Vašíčková et al. (2018) found that music students’ stress levels varied depending on their roles, with artists experiencing higher stress during solo performances and music managers facing more stress in group settings. This suggests that factors other than self-concept might also play significant roles in mediating stress and flourishing. Furthermore, Kuebel (2019) emphasized the need for increased awareness and utilization of self-care strategies among music students and educators, indicating that self-concept alone may not be sufficient to address academic stress effectively. Comprehensive approaches that include self-care practices might be necessary to enhance flourishing among music students.

### Indirect effects through emotional-spiritual self-concept

Overall, our findings reveal that academic stress has significant indirect effects on music students’ flourishing through their emotional and spiritual self-concept. Aligned with our evidence, previous studies support the notion that self-concept, particularly in emotional and spiritual domains, plays a crucial role in mediating the relationship between academic stress and flourishing among music students. For instance, McConkey and Kuebel (2022) highlighted that emotional competence skills are vital for managing stress among music education students. Their research underscores the importance of developing an emotional self-concept to handle academic stress better. Additionally, Qi (2023) demonstrated that music therapy plays a significant role in relieving negative emotional stress and promoting mental health among university graduates. This further supports the idea that emotional self-concept can be an effective buffer against academic stress. While many studies agree on the importance of emotional and spiritual self-concept in managing academic stress, some research presents different perspectives. Kamza and Bazarbekova (2023) noted that while classical and modern music positively impacted students’ psycho-emotional state, rock music had a negative effect. This indicates that the type of music and individual preferences might also influence the relationship between academic stress and flourishing beyond self-concept.

### Combined mediator effects

Finally, the findings of the present study reveal that academic stress has significant indirect effects on music students’ flourishing through the combined mediating effects of musical self-concept communication and musical self-concept ability and ambition, as well as musical self-concept emotion and musical self-concept spirituality. Although little empirical research has been conducted on this specific type of relationship, some contrary evidence needs to be acknowledged. The mediation analysis revealed that the Emotional and Spiritual dimensions (M3 and M4) had the strongest indirect effects, significantly attenuating the negative influence of academic stress on flourishing. The Communicative dimension (M1) also showed a significant but smaller mediating effect. These findings suggest that internalized emotional and spiritual engagement with music, and to some extent communicative musical expression, may serve as effective buffers against the psychological burdens of academic pressure among music students. While Emotional and Spiritual self-concept dimensions significantly mediated the relationship between academic stress and flourishing, the Ability and Ambition dimension (M2), as well as its combined pathway with Communication (M1), did not. Thus, Hypotheses H3 and H6 were not supported. This may suggest that more extrinsically focused facets of musical self-concept—centered on achievement and technical skill—do not effectively protect against stress in the same way that emotionally resonant or spiritually fulfilling engagement with music does. It is also possible that ambition in music performance is itself a source of internal pressure rather than relief. Hyo-rim and In-su (2020) discussed the resilience of musically gifted students who experienced growth in stressful private lessons, suggesting that individual resilience and coping strategies might also play significant roles in influencing the outcomes. This highlights the complexity of the relationship between academic stress and flourishing, suggesting that multiple factors, including self-concept, resilience, and coping strategies, interact to shape students’ well-being. Although not explicitly hypothesized, the total indirect effect of musical self-concept was statistically significant (*β* = −0.077). This suggests that, taken as a whole, the multidimensional construct of musical self-concept significantly attenuates the negative impact of academic stress on flourishing. We interpret this exploratory result as evidence that while not all dimensions individually mediate this relationship, their combined influence is meaningful. This supports our conceptualization of musical self-concept as an embodied and integrative construct, with holistic implications for well-being in music education.

Overall, these findings underscore that the emotional and spiritual aspects of musical self-concept, when combined, significantly mediate the relationship between academic stress and flourishing. In contrast, the instrumental dimensions did not show a statistically significant mediating effect. This finding aligns with prior research on general student populations, which has shown that students with higher self-concept and emotional engagement tend to flourish more. However, the applicability of these findings to music students remains uncertain, given the unique stressors and identity dynamics in music education.

### Limitations of the present study and suggestions for future research

Despite this study’s strengths and insights, several limitations must be acknowledged. Firstly, the cross-sectional design limits the ability to infer causality between academic stress and flourishing through the mediators of musical self-concept dimensions. Longitudinal studies are needed to establish temporal relationships and causative effects more conclusively.

Secondly, the reliance on self-report measures, such as the Educational Stress Scale for Adolescents (ESSA) and the Musical Self-Concept Inquiry (MUSCI), may introduce response biases. Participants might overestimate or underestimate their levels of stress and self-concept due to social desirability or inaccurate self-assessment. Incorporating objective measures, such as physiological stress indicators or performance assessments, could provide a more comprehensive understanding of these variables.

The sample composition presents another limitation. However, the robust sample size of 1,767 Chinese university music students may not generalize to music students in other cultural contexts. Cultural factors can significantly influence stress perceptions and self-concept; therefore, cross-cultural studies are recommended to validate and extend the findings beyond the Chinese context.

Additionally, the study’s focus on university students excludes other populations that might experience different dynamics between academic stress and flourishing, such as professional musicians, younger students, or amateur musicians. Future research should consider these diverse groups to provide a more holistic view of the relationship between academic stress, self-concept, and flourishing in music.

Lastly, while the shortened scales used for measuring musical self-concept provided adequate reliability, the lower Cronbach’s Alpha values for the Spirituality, Ability, and Ambition dimensions (0.69) suggest that these measures may benefit from further refinement and validation to ensure they accurately capture the intended constructs.

Future research should address the limitations outlined above to build on the current study’s findings. Longitudinal studies would be particularly valuable in establishing causality and examining how the relationships between academic stress, self-concept dimensions, and flourishing evolve. Such studies could also identify critical periods when interventions might be most effective.

To mitigate response biases inherent in self-report measures, future studies should incorporate a mixed-methods approach. Combining quantitative self-reports with qualitative interviews and objective measures (e.g., cortisol levels, academic performance) could yield richer, more nuanced data.

Expanding the sample to include music students from diverse cultural backgrounds will enhance the generalizability of the findings. Comparative studies across different countries and educational systems could uncover cultural nuances and provide insights into universal versus culture-specific aspects of the relationships studied.

Research should also explore the experiences of different populations within the music community, such as professional musicians, pre-university students, and amateur musicians. Understanding how academic stress and self-concept interact in these groups could inform tailored interventions for each demographic.

Further validation and refinement of the MUSCI, especially the Spirituality, Ability, and Ambition dimensions, are necessary. Future studies should test the psychometric properties of these scales in larger and more diverse samples to enhance their reliability and validity.

Lastly, intervention studies are crucial. Investigating the effectiveness of specific interventions, such as mindfulness programs, music therapy, or peer support groups, in enhancing musical self-concept and reducing academic stress could provide practical solutions for improving the well-being and flourishing of music students. Future research could consider randomized controlled trials (RCTs) as one rigorous approach to test intervention efficacy. However, given the complexity of constructs like musical self-concept and flourishing, mixed-methods designs may offer richer insights by capturing both causal effects and subjective experiences. In summary, while this study provides valuable insights into the indirect effects of academic stress on music students’ flourishing through musical self-concept dimensions, addressing its limitations and expanding future research will deepen our understanding and enhance support strategies for this unique population.

### Implications for music university teachers and teaching innovations

The findings of this study underscore the critical role of musical self-concept in mediating the relationship between academic stress and flourishing among music students. For music university teachers, this highlights the importance of integrating self-concept development into the curriculum (Tu, 2022). By designing courses and activities that promote self-awareness, self-efficacy, and emotional and spiritual engagement with music, educators can help students build a stronger, more positive self-concept. This, in turn, can mitigate the negative effects of academic stress and enhance overall wellbeing (Degé and Schwarzer, 2018).

Music students often face unique stressors related to performance, workload, and high expectations (Pang et al., 2024). To address these challenges, universities with music programs should implement support systems tailored to the specific stressors of music students, such as performance anxiety, competitive evaluation, and identity-related pressure. In addition to general counseling services and stress management workshops, specialized interventions like music performance coaching, mindfulness programs focused on pre-recital stress, and peer-led reflection groups may be particularly effective. Teachers in music departments can also benefit from professional development sessions on mental health literacy and emotional communication in conservatory settings, enabling them to recognize distress and respond with appropriate support or referrals (Song et al., 2019). Fostering a culture of openness around psychological challenges within music faculties is essential for promoting flourishing. A supportive environment from instructors may help nurture students’ musical self-concept and well-being. Drawing on Self-Determination Theory (Deci and Ryan, 2000), we propose that autonomy-supportive teaching, along with practices that foster competence and relatedness, can enhance students’ intrinsic motivation and reinforce a positive, embodied musical self-concept. These psychologically supportive conditions are likely foundational to student flourishing in higher music education.

The study’s findings on the importance of emotional and spiritual self-concept suggest that music education should focus not only on technical proficiency but also on the emotional and spiritual dimensions of musical engagement. Teachers can encourage students to explore and express their emotions through music, providing opportunities for reflection and discussion on how music impacts their emotional and spiritual well-being. Incorporating practices such as mindfulness, meditation, and discussions on the spiritual aspects of music can enrich students’ educational experiences and support their holistic development. Flourishing should therefore be viewed as a central objective in music education—a view supported by recent work such as Ascenso et al. (2016) highlight the importance of well-being and self-concept as core outcomes of professional musical development.

The significance of musical self-concept communication in mediating the effects of academic stress implies that fostering a collaborative learning environment can be beneficial. Teachers should encourage group activities, ensemble performances, and peer-to-peer learning opportunities. By promoting collaboration and communication, students can develop their ability to connect with others through music, enhancing their sense of belonging and reducing feelings of isolation and stress.

Given the study’s successful use of online receptive music experiences to reduce stress and anxiety, music university teachers should consider incorporating technology-based interventions into their teaching practices (Yang, 2024). Virtual music therapy sessions, online relaxation music playlists, and digital tools for self-assessment and stress management can be integrated into the curriculum to provide students with accessible resources for managing stress (De Bruin and Merrick, 2023).

To effectively support students’ self-concept and well-being, music university teachers need ongoing professional development (Sun-Ah et al., 2019). Training in areas such as mental health awareness, stress management techniques, and innovative pedagogical approaches can equip teachers with the skills and knowledge necessary to address students’ needs comprehensively. Workshops, seminars, and collaborative learning opportunities for teachers can foster a culture of continuous improvement and innovation in music education (Young, 2017).

Universities should encourage research and innovation in music education to improve teaching practices and continuously support student well-being (Parkyun and Jeong, 2020). Teachers can be involved in action research projects to explore new methods and interventions that enhance self-concept and reduce academic stress. Sharing successful strategies and findings through academic publications and professional networks can contribute to the broader field of music education.

## Conclusion

The present study demonstrates that academic stress negatively affects flourishing among university music students, and that this relationship is mediated by students’ musical self-concept, particularly in its emotional-spiritual and instrumental dimensions. These findings reinforce the importance of embodiment, not just as a conceptual frame but as a measurable and meaningful mechanism through which music students navigate academic demands and sustain psychological flourishing.

The implications for music university teachers and teaching innovations are significant. By focusing on enhancing students’ self-concept across emotional, spiritual, communicative, and ambitious dimensions, educators can create more supportive and enriching learning environments. Strategies such as comprehensive support systems, collaborative and reflective practices, use of technology, and professional development are key to fostering resilience and wellbeing.

These results contribute to a growing body of research on music education and student mental health, emphasizing the need for pedagogical approaches that recognize the cognitive, emotional, and embodied complexity of musical training. Future studies could explore these mediating mechanisms in different cultural contexts and educational settings, as well as the longitudinal effects of targeted interventions to strengthen musical self-concept and reduce academic stress.

## Data Availability

The raw data supporting the conclusions of this article will be made available by the authors, without undue reservation.
